# Frailty effects in networks: comparison and identification of individual heterogeneity *versus* preferential attachment in evolving networks

**DOI:** 10.1111/j.1467-9876.2010.00746.x

**Published:** 2011-03

**Authors:** Birgitte Freiesleben de Blasio, Taral Guldahl Seierstad, Odd O Aalen

**Affiliations:** University of OsloNorway

**Keywords:** Citation network, Frailty, Network model, Power law network, Preferential attachment, Sexual partner network, Yule distribution

## Abstract

Preferential attachment is a proportionate growth process in networks, where nodes receive new links in proportion to their current degree. Preferential attachment is a popular generative mechanism to explain the widespread observation of power-law-distributed networks. An alternative explanation for the phenomenon is a randomly grown network with large individual variation in growth rates among the nodes (frailty). We derive analytically the distribution of individual rates, which will reproduce the connectivity distribution that is obtained from a general preferential attachment process (Yule process), and the structural differences between the two types of graphs are examined by simulations. We present a statistical test to distinguish the two generative mechanisms from each other and we apply the test to both simulated data and two real data sets of scientific citation and sexual partner networks. The findings from the latter analyses argue for frailty effects as an important mechanism underlying the dynamics of complex networks.

## 1. Introduction

Our aim in this paper is to study the mechanisms behind the growth of large networks. The models that we consider are random-graph processes where nodes and links are added to the graph, but without removal of either nodes or links.

In recent years, empirical data on large-scale networks have become available and have provided valuable insights into the structure of real world connected systems. In many complex networks it is found that the degrees of the nodes follow a power law distribution. In an early reference in this field de Solla Price (1976) found that the number of citations of scientific articles follows a power law, and he showed that this power law can be explained if one assumes that the probability that a new publication cites a given article is proportional to the number of citations. This was taken up by Barabási and Albert (1999), who proposed a similar model for the World Wide Web, and who coined the name ‘preferential attachment’ for this mechanism. Today, most existing models that are aimed at reproducing power law graphs incorporate a preferential attachment mechanism of some kind; numerous examples can be found in Dorogovtsev and Mendes (2003). However, already in 1925, Yule had published a closely related stochastic branching model. The Yule process is general and has been adapted to networks; see Krapivsky *et al.* (2000), Newman (2005) and Dorogovtsev *et al.* (2000).

Jeong *et al.* (2003) proposed a method to identify a preferential attachment process. The idea is to estimate, for every *k*, the intensity Π(*k*) by which nodes of degree *k* acquire new links during a small time interval Δ*t*. The preferential attachment hypothesis predicts that the rate Π(*k*) is a monotonically increasing function of *k*, and Jeong *et al.* (2003) found that this is indeed so for several real world networks. In other words there is a correlation between a node's attractiveness and its popularity, where we interpret the *attractiveness* of a node as its rate of acquiring new links, and the *popularity* of a node as the number of links that it has already acquired.

However, the preferential attachment hypothesis actually makes a stronger prediction, namely that a node is attractive *because* it is popular, i.e. a node with high degree attracts new links at a higher rate than other nodes *because* it has high degree. Despite the popularity of the preferential attachment hypothesis in explaining the structure of complex networks, it seems natural also to investigate the opposite relationship: a node may have some intrinsic quality causing it to acquire links at a higher rate than other nodes. Such nodes will in general end up with higher degree than less attractive nodes; thus, in this setting we shall also find an increasing Π(*k*), although the growth mechanism is different from preferential attachment.

In some networks a frailty mechanism provides a seemingly reasonable explanation of the connectivity distribution. For example in a sexual network the degree of a node (i.e. the number of previous sexual partners) is not displayed and cannot be used directly as a selection criterion. Likewise, research papers with a high citation count are likely to have some merit, and are, one would hope, cited again because of their quality, and not merely because of their previous popularity. However, papers with many citations are more likely to be found in a literature search by a potential citer and are also for this reason more likely to be cited. It seems reasonable that in many real networks there is a mixed mechanism, such that a node's attractiveness increases with its popularity, but such that there still is heterogeneity between the nodes, which is not directly related to their degrees.

In this paper we shall study a random-graph model which evolves by a mechanism which mimics this behaviour, and we shall investigate statistical methods by which we can distinguish such a process from a pure preferential attachment process. The random-graph model that we propose we call a *frailty graph*. At birth every node *v* is assigned a *frailty Z*_*v*_, which is distributed according to some probability distribution *Z*. Frailty is a term that is used in event history analysis to describe unobserved heterogeneity in data. The random graph then grows in such a way that existing nodes acquire new links with a rate that is proportional to its assigned frailty variable. We shall assume that we cannot observe the frailty *Z*_*v*_ for a given node *v*; however, it is possible to infer properties about the distribution of *Z* from the behaviour of the random-graph process.

We can consider a preferential attachment process and the frailty graph process as two extremes: in the preferential attachment process nodes are equal at the outset, and their rate of acquiring new links depends solely on their degree. Nodes which enter the graph at a later time, or nodes that are unlucky in the beginning and acquire few links, will have little chance of overtaking nodes with an early success. In the frailty graph process, however, there is an inherent unevenness between the nodes from the beginning and, even if a node with unfavourable frailty is lucky and receives several links in one time period, this will not affect its ability to attract new links in another time period. Furthermore, entering the graph at a later time is not an obstacle to becoming a successful node, as long as the node has a favourable frailty variable.

The method to identify preferential attachment, which was proposed by Jeong *et al.* (2003), considers the degree distribution of the network at two relatively close observation times and estimates the link acquiring rate of a node as a function of its degree. As explained above, this method does not distinguish a network evolving by the preferential attachment mechanism from a frailty network. As an alternative we propose methods which make use of three observation times, *t*_1_, *t*_2_ and *t*_3_, or two observation times combined with information on age.

A neat property of the preferential attachment mechanism is that it automatically leads to graphs where the degrees follow a power law distribution, provided that the rate with which a node acquires new links is growing linearly with its degree. A power law distribution has been observed in many real world complex networks; for this reason the preferential attachment hypothesis is an appealing explanation. In the frailty model we obtain a power law distribution only if the frailty variable itself is power law distributed; thus, a power law may be more difficult to rationalize if we assume that a frailty process is the underlying mechanism of the network. However, power laws are observed in many situations in the real world, e.g. in social interactions as pointed out by Zipf (1949), and may be caused by several mechanisms (Newman, 2005), so it is not so far fetched to assume that such a frailty variable may have a power law distribution. In this paper we derive a distribution for the frailty variable which ensures that the graph process asymptotically has the same degree distribution as the Yule process and thus follows a power law.

We shall also consider two real world networks, namely citation data for a certain set of mathematical research papers, and the sexual network of a subset of the Norwegian population. Much work has been done on the study of sexual networks, since this is relevant with regard to the spread of sexually transmitted infections. In a study of homosexual men attending a sexually transmitted infections clinic, Colgate *et al.* (1989) observed that the sexual contacts follow a power law degree distribution. This finding was supported by Liljeros *et al.* (2001), on the basis of analyses of data from a population-based sexual survey in Sweden, and later Liljeros *et al.* (2003) suggested that preferential attachment could be of relevance for sexual network growth. However, these issues have been the subject of some controversy, and existing data samples are too small to verify scaling behaviour for more than 1–2 decades. In an attempt to find growth models for sexual networks, Jones and Handcock (2003) and Handcock and Jones (2004) noted that heterogeneity between the nodes for forming new links is the mechanism that is best supported by data, and they concluded that

‘a unitary behavioural process, such as preferential attachment, is unlikely to underlie empirical sexual network degree distributions’.

Moreover, they found that, although the sexual network in some portions of some societies are well fitted by a power law, a better fit is generally obtained by a negative binomial distribution and variants thereof. Similarly, Hamilton *et al.* (2008) found that, when compared with alternative models, the power law hypothesis fails to have consistent support. In a different setting, Stephen and Toubia (2009) compared preferential attachment with other possible mechanisms to find an explanation for the process of link addition in a certain social commerce network. They found that the mechanism which fits the data best is a mechanism that is based on vertex attributes, akin to a frailty effect.

Real networks are governed by complex social, behavioural and evolutionary dynamics and the frailty and preferential attachment graph models that are considered here are idealizations that involve severe reduction of that complexity. It is probable that empirical networks may evolve by a combination of the two mechanisms in addition to time inhomogeneous growth. In the first part of the paper we focus on the two ‘pure’ graph models and investigate the theoretical differences between them; then we shall consider some data examples and discuss how they relate to the two models.

## 2. Frailty power law distribution

Our objective is to compare frailty networks with networks evolving by preferential attachment. For this, we need to find the probability distribution of the frailty variable *Z*, which ensures that the random frailty graph has the same asymptotic behaviour as the preferential attachment graph. As already mentioned, networks growing by the preferential attachment mechanism automatically have a degree distribution which is asymptotically scale free, provided that the ‘preference function’Π(*k*) is linear in *k*. The example that we shall consider here is a general preferential attachment process which was proposed by Yule (1925).

In this section, we start by deriving analytically the probability distribution of the frailty variable *Z*, which ensures that the frailty graph is asymptotically similar to the Yule graph, and we shall consider finite size effects of the scaling function. In the subsequent sections we provide methods for testing for preferential attachment in networks, and we compare the two types of graphs by using simulations. Lastly, we shall apply the test to real data in terms of citation and sexual partner networks.

## 2.1. Graph models

The implementation of the graph processes in the simulations is as follows: we start with some fixed graph *G*_0_ and let *G*_*m*_ be the graph after *m* steps in the process. To every node *v* in *G*_*m*_, we associate a probability *p*_*v*,*m*_, such that, for every *m*≥0, Σ_*v* ∈ *G*_*m*__ *p*_*v*,*m*_=1. In the Yule process the probability that is associated with a node is proportional to its degree; in the frailty process, it is proportional to an unobserved frailty variable that is assigned to the node at birth.

At every step in the graph process, one of the following two actions is performed. With probability *p*_node_, a new node is added to the graph. It is then attached to one of the existing nodes, in such a way that every node *v* has probability *p*_*v*,*m*_ of being chosen. With probability *p*_link_=1−*p*_node_ a new link is added between existing nodes. The origin is chosen uniformly at random; the target node is chosen according to the probabilities *p*_*v*,*m*_.

The difference between the Yule process and the frailty process is the way in which the probabilities *p*_*v*,*m*_ are calculated. Let *d*_*v*,*m*_ be the degree of *v* in *G*_*m*_, and let *s*_*m*_ be the total number of links in the graph. Then, in the Yule process, *p*_*v*,*m*_=*d*_*v*,*m*_/2*s*_*m*_. In the frailty process, whenever a node arrives it is assigned a *frailty* value *z*_*v*_ which is chosen according to the probability distribution of a given positive random variable *Z*. The probabilities *p*_*v*,*m*_ are then given by the equation *p*_*v*,*m*_=*z*_*v*_/Σ_*w* ∈ *G*_*m*__ *z*_*w*_.

Thus, in the Yule process, a node is chosen with probability proportional to its degree, whereas, in the frailty process, a node is chosen with probability proportional to its frailty value.

We let *p*_link_=*h*/(*h*+1) and *p*_node_=1/(*h*+1), where *h* is a parameter indicating the expected number of links added between the addition of two consecutive nodes.

## 2.2. Yule distribution

The Yule distribution has its origin in a mutational evolutionary model; see Simon (1955), Willis (1922) and Yule (1925). It has been adapted to networks in different ways; here we follow the derivation by Newman (2005). Consider a growing network where new nodes arrive consecutively at each time step. At the point of arrival, new nodes make *j*_0_ links to existing nodes, where *j*_0_ may take the values *j*_0_=0,1,2,. If *j*_0_=0, the model requires an additional ‘attractiveness’ parameter *a*>0 for the new nodes to engage in the preferential attachment process. Between the arrival of new nodes, a constant of *m* links are formed between the existing nodes; *m* may take the values *m*=0,1,2,…, though either *j*_0_ or *m* must be a positive integer for links to form. Here the target ends are chosen according to their present degree *j*_*i*_ with *p*_*i*_=*j*_*i*_/Σ_*k*_ *j*_*k*_. In principle, at time *t*=0 the system starts with two nodes connected by *m* links, so at time *t*=*t*^′^ there are *m*+(*m*+*j*_0_)*t*^′^ links in a system of size *N*=2+*t*^′^ nodes. Newman (2005) has shown with the use of a master equation technique that the asymptotic distribution of the degree *J* of a randomly chosen node is given by


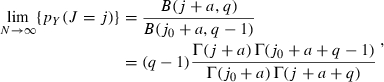
(1)

where *q*=2+(*j*_0_+*a*)/*m* is the scaling constant


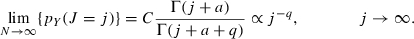
(2)

The Barabási–Albert preferential attachment model where new nodes enter and link to *m* existing nodes chosen with linear preference arises as a special case for *m*=*a*;*j*_0_=0 and has the scaling property *p*(*j*)∝*j*^−3^.

## 2.3. Frailty graph

We aim to find the frailty probability density function (PDF) of a randomly grown graph that has a probability distribution function that is similar to the Yule graph equation (2). For this we note that the degree of any single node is a random variable which is asymptotically Poisson distributed. We first seek an expression for the probability-generating function of the degrees of the random graph. Then we can recover the frailty rate distribution from the inverse Laplace transform of the generating function.

We consider a random graph with *N* nodes, where *N*→∞. We assume that the *i*th node is assigned a frailty variable *Z*_*i*_, where *Z*_1_,*Z*_2_,…,*Z*_*N*_ are independent random variables which have the same distribution as a given positive random variable *Z*. The expected degree of a given node *i* is proportional to its frailty variable *Z*_*i*_; however, it also depends on the age of the node, as older nodes will generally have a larger degree than younger nodes. Below we shall define a random variable *Y* which is a ‘time-adjusted’ frailty variable, taking account of both the inherent frailty variable of a node and its age.

After *m* steps of the process, the sum of the frailty variables of all the nodes in the graph will be roughly *mμp*_node_, where 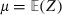. When we consider the asymptotic case, the error in this approximation is of lower order *O*(*m*) and can be ignored. If node *i* was added at step *m*_*i*_, then the degree of *i* after *m* steps will be approximately Poisson distributed with mean


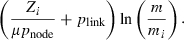


Let 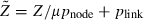 and *γ*_*m*_(*m*_*i*_)= ln (*m*/*m*_*i*_), and let 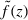 be the PDF of 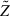.

Now, suppose that we pick a node *v* uniformly at random from the graph, and assume that it is a node that is not included in the initial graph *G*_0_. The birth time of *v* is then a random variable *M*, uniformly distributed on the interval [*m*_0_,*m*_1_]. Then the degree of *v* will be Poisson distributed with mean 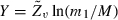. Let *g*(*z*) be the PDF of *Y*. By involved, but standard, calculations, we can show that *g*(*z*) is related to *f*(*z*) by


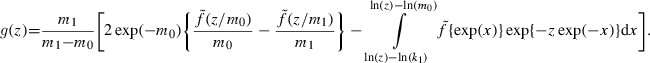
(3)

Unfortunately it is not possible to express 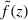 as a function of *g*(*z*) in closed form, but it is possible to calculate 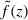 numerically, by using equation (3). The PDF of *Z* is then given by 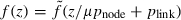.

The function *g*(*z*) is approximately a ‘stretched-out’ version of 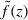, so *Y* has somewhat higher variability than *Z*, but it behaves similarly in many important ways. In particular, we can show that, if *f*(*z*) follows a power law, then 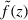 also follows a power law, with the same exponent. We shall use *g*(*z*) as an approximation for the PDF of the frailty variable, rather than the more correct *f*(*z*), in the following sections. This eases the calculations, and the approximation is sufficiently good to illustrate the similarities of the Yule graph and the frailty graph.

## 2.4. Generating function of the frailty graph

As a first step, consider a random graph with *N* nodes, and let *N*→∞. The graph has an arbitrary degree distribution described by *P*(*j*) on the non-negative integers *j*=0,1,…. Let *Y* be a random variable and let *g*(*z*) be the density function of *Y*. Moreover, let *Y*_1_,…,*Y*_*N*_ be independent random variables with the same distribution as *Y*. We assume that the degree of the *i*th node is a Poisson-distributed random variable with mean *Y*_*i*_. Then *P*(*j*) and the Laplace transform *L*_*g*_ of the rate distribution *g*(*z*) are related by the equation


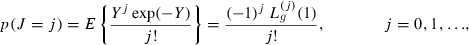
(4)

where 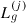 is the *j*th derivate of the Laplace transform *L*_*g*_. The probability-generating function *G*(*s*) of equation (4) has a power series representation with values of *P*(*j*) as the coefficients. With use of equation (4) we find that


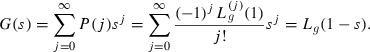


From the above equations we see that the probability-generating function of a Poisson distribution with random rate parameter *Y* has a simple relationship to the Laplace transform of the rate distribution


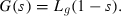


Now we set this random probability distribution function equal to the Yule distribution, *P*(*j*)=*p*_Yule_(*j*). Then we can obtain the rate distribution from the inverse Laplace transform 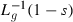. Inserting equation (1) we find that the expression takes the form


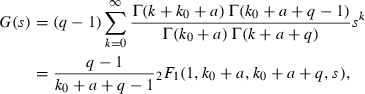


where _2_*F*_1_ is the Gauss hypergeometric function; see equation 15.1.1 of Abramowitz and Stegun (1984). In this equation we have reparameterized the Yule probability distribution *j*→*k*+*k*_0_ for the summation to start at *k*=0. With this result we can express the Laplace transform by


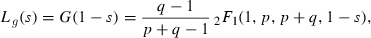
(5)

where we have substituted *p*=*k*_0_+*a*. The constant *p* is identical to the initial proportionality factor for new nodes in the Yule process. In the simulations we use initial attractiveness *p* ∈ (0,1] and scaling constants *q* ∈ [2,3].

## 2.5. Frailty distribution

Because of results by Abate and Whitt (1999a,b);, the inverse Laplace transform of equation (5) is found to be a *beta mixture of exponential* distributions that is averaged with respect to the beta distribution. Abate and Whitt (1999a, b) also showed that both the Gauss hypergeometric function and the beta mixture of exponential distributions can be expressed by continued fraction representations in terms of Laguerre series, which may conveniently be used for numerical inversion of the Laplace transform. The beta mixture of exponential distributions is identical to the frailty rate PDF, and we may write


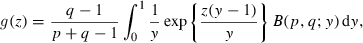
(6)

where *B*(*p*,*q*;*y*) is the standard beta distribution


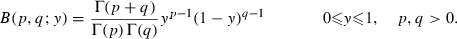


To solve the integral in equation (6) we make a substitution of the variable *x*=(1−*y*)/*y* and rewrite the frailty PDF in the form


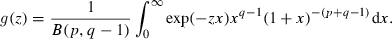
(7)

Then it follows from equation 13.2.5 of Abramowitz and Stegun (1984) that the integral has the solution


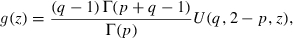
(8)

where *U*(·) is the Tricomi hypergeometric function, which is also known as the confluent hypergeometric function of the second kind. Alternatively, using equation 13.1.3 of Abramowitz and Stegun (1984) we find that the function can be written as a combination of regularized confluent hypergeometric functions of the first kind, 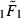 (Kummer's function),


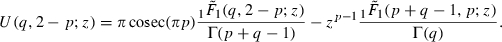


To make a frailty graph, we draw each frailty independently from the present distribution. A randomly grown graph with frailty *Z* that is obtained in this way will have the desired scaling property of equation (2).

## 2.6. Scaling of the frailty distribution

The asymptotic behaviour of *g*(*z*) for *s*→∞ can be obtained from Tauberian theory by studying the behaviour of its Laplace transform as *s*→0. From properties of the Gauss hypergeometric function that are described in Abramowitz and Stegun (1984) it follows that *L*_*g*_(*s*) is of the limiting form *L*_*g*_(*s*)∼*s*^*q*−1^. Using theorem II in section XIII.5 of Feller (1968) we find that the scaling property of *g*(*z*) is


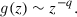


This result is in line with the general finding that combinations of exponentials like equation (6) result in a power law distribution. This has been studied by Miller (1957) in the context of frequencies of words in a text, and by Reed and Hughes (2002) who considered processes of exponential growth, which have exponentially distributed survival times.

## 2.7. Finite size systems

The rate frailty model equation (8) approaches the Yule distribution asymptotically. However, empirical networks have finite sizes and, hence, their scaling behaviour is confined to a limited regime. Hence, to compare the frailty and the Yule growth processes, we need to find the approximate frailty distribution that has equivalent scaling behaviour as a Yule-distributed network with a finite number of nodes. As before we shall use the frailty PDF *g*(*z*) as an approximation for the real frailty variable, rather than applying the transform (4).

Yule (1925) generalized the Yule process to a situation with a finite time horizon. He showed that the Yule probability distribution in this case is found by replacing the beta functions in equation (1) by incomplete beta functions. Hence


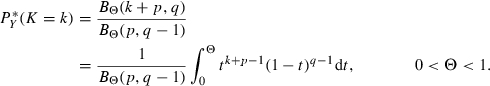


We use the notation 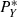 to designate the time-limited Yule distribution. The truncation has the effect of introducing an exponential cut-off on the scaling at *k*_cut_∼1/(1−*Θ*); see Dorogovtsev and Mendes (2003). Thus, in this case we approximate the Yule probability distribution by


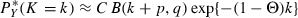
(9)

where *C* is a normalization constant given by


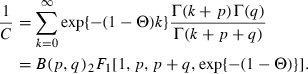


We follow the same procedure as in the previous section. The generating function of equation (9) takes the form


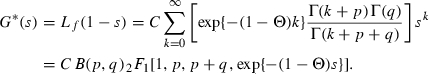


To find the inverse Laplace transform, we note that, in the limit *Θ*→1, we have exp {−(1−*Θ*)}∼*Θ*+*O*(1−*Θ*)^2^. In this case we obtain the generating function


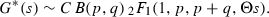
(10)

From equation (10) it is seen that the scale on the Yule process is carried over to the frailty variable. Using the results from the previous section, equation (6) and equation (7), the finite size frailty distribution may be approximated by


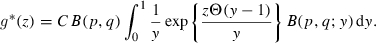
(11)

There is a simple way to obtain the desired frailties instead of using the Tricomi hypergeometric function. From equation (1.22) in Abate and Whitt (1999b) it is seen that the frailty PDF (6) is a second beta mixture of exponential distributions. This function is obtained from the random variable *Y*_2_=*Y*/(1−*Y*) where *Y* has a standard beta PDF with parameters (*p*,*q*−1). As noted by Abate and Whitt (1999b), page 4, beta exponential mixtures can be generated as products of independent random variables. Let *X* and *Y*_2_(*p*,*q*) be random variables with PDFs exp (−*y*) and *B*_2_(*p*,*q*−1;*y*), i.e. a beta PDF of the second kind. Then the second mixture of exponential distributions PDF can be represented via the random variable *Z*_2_=*X* *Y*_2_(*p*,*q*−1).

These properties may conveniently be used for generating a frailty PDF giving a random graph as in equation (6). First, we generate a standard beta PDF *Y*=*B*(*p*,*q*−1). Second, we make the *Y*_2_=*B*_2_(*p*,*q*−1) random variable as *Y*_2_=*Y*/(1−*Y*). Third, we generate a unit exponential random variable *X*= exp (−*y*). From these variables we construct a random variable *Z*_2_=*XY*_2_. This new variable *Z*_2_ will have the required form, since


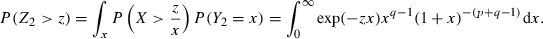


For a finite size system, what remains is to scale the random variable *Z*_2_/*Θ* on the basis of the characteristic network size *N* and the scale factor *q* of the Yule network.

## 3. Preferential attachment test

A test for preferential attachment ought to be able to distinguish the two random-graph processes that were presented in the previous section, namely the Yule and the frailty process. In Jeong *et al.* (2003) it was suggested to identify a preferential attachment process by verifying that the rate Π(*k*) is a monotonely increasing function of *k*, in the following manner. Let *t*_1_ and *t*_2_ be two points in time, and let *G*_*t*_1__ and *G*_*t*_2__ be the graphs at the given points of time. For a given node *v*, which is present in the graph at both times, let *D*_1_=*D*_1_(*v*) be its degree in *G*_*t*_1__ and *D*_2_=*D*_2_(*v*) be its degree in *G*_*t*_2__. We shall consider *D*_1_ and *D*_2_ to be random variables, and we let *X*_1_=*X*_1_(*v*)=*D*_2_−*D*_1_ be the increase in *v*’s degree from time *t*_1_ to *t*_2_. We estimate how *X*_1_ depends on *D*_1_. Jeong *et al.* (2003) found that in several networks the expectation of *X*_1_ depends linearly on *D*_1_. To estimate this dependence, the times *t*_1_ and *t*_2_ should be chosen quite close to each other.

**Fig. 1:**
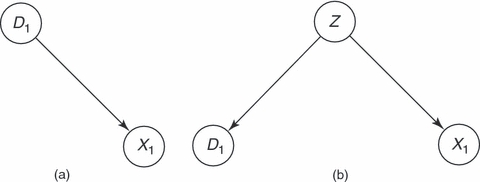
In (a) a preferential attachment process, the number *X*_1_ of new links that a node receives in a given time interval is directly affected by the node's degree *D*_1_ at the beginning of the interval: in (b) a frailty process, *D*_1_ and *X*_1_ do not affect each other directly but are both affected by a common cause, namely the node's frailty variable *Z*.

However, this procedure is not sufficient to distinguish between the Yule network and the frailty network. To see this, it is convenient to visualize the dependences between *X*_1_ and *D*_1_ with a so-called causal diagram (Pearl, 2000); Fig. 1. In a causal graph the relevant random variables are represented by nodes (*x*,*y*,…) and an arc *x*→*y* indicates that *x* has a causal effect on *y*. As we can see, *D*_1_ and *X*_1_ are positively correlated in both the preferential attachment process and the frailty process: in the former process they are correlated because *D*_1_ has a direct effect on *X*_1_, whereas in the latter they are correlated because they share a common ancestor, namely *Z*, which acts as a confounder. Hence a procedure merely measuring the correlation between *X*_1_ and *D*_1_ will not be able to distinguish the two processes.

Instead, we shall assume that we can measure the graph at three time points, *t*_1_, *t*_2_ and *t*_3_. For *i*=1,2,3 we let *G*_*t*_*i*__ be the graph at time *t*_*i*_ and, for a given node *v*, the degree of *v* in *G*_*t*_*i*__ is denoted by *D*_*i*_=*D*_*i*_(*v*). We let *X*_1_=*X*_1_(*v*)=*D*_2_−*D*_1_ and *X*_2_=*X*_2_(*v*)=*D*_3_−*D*_2_.

**Fig. 2:**
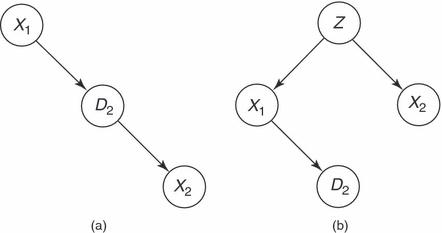
In both (a) a preferential attachment process and (b) a frailty process the number *X*_1_ of links that a node acquires in the first time interval affects the node's degree *D*_2_ at the end of that interval: in a preferential attachment process *D*_2_ in turn affects the number *X*_2_ of links that are acquired in the next time interval, but there is no causal relationship between *X*_1_ and *X*_2_, apart from that going through *D*_2_; in a frailty process there is no direct relationship between *D*_2_ and *X*_2_, but both *X*_1_ and *X*_2_ are affected by a common cause *Z* (*D*_1_ is not displayed since it no longer plays a part in the analysis)

In Fig. 2, we see the causal diagram in this situation, including the ‘new’ variables *D*_2_ and *X*_2_. Let us now see what happens if we perform a statistical analysis controlling for *D*_2_. In the preferential attachment process, controlling for *D*_2_ clearly blocks the only path between *X*_1_ and *X*_2_. Thus, this will remove the correlation between the two variables. In the frailty process, the path between *X*_1_ and *X*_2_ is still intact after controlling for *D*_2_. In this case, the correlation between *X*_1_ and *X*_2_ is therefore still present.

Hence, estimating the correlation between *X*_1_ and *X*_2_ when controlling for *D*_2_ will distinguish the preferential attachment process from the frailty process.

One way that this can be done is by doing linear regression, where both *X*_1_ and *D*_2_ are covariates, and a transformation of *X*_2_ is the dependent variable, i.e. we regress to find the parameters *β*_0_, *β*_1_ and *β*_2_ such that


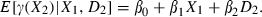
(12)

We then check to see whether the parameter *β*_1_ is significantly different from 0. If it is, then we have an argument in favour of the frailty process; if not, the argument is in favour of the preferential attachment hypothesis. The transformation *γ* must be chosen so that the assumptions underlying linear regression are satisfied to a reasonable degree. In a simulation of the Yule process, and in the case of the citation data, we find that a Box–Cox transform with parameter 0.5 is suitable. For the sexual data, it turns out that a Box–Cox transform with negative parameters is appropriate.

This method is particularly suitable in the cases where Π(*k*) has been estimated to be linear in *k*. Otherwise, if we have been able to estimate the functional form of Π(*k*), we might exchange *D*_2_ with Π(*D*_2_) in the above regression.

The method that was described above requires that the graph be observed at three separate occasions. However, in many cases the nodes may have a well-defined birth time, which is known to us. We can then use this as one of the time points, and we thus require only two further observation points. In this case we let *t*_1_ and *t*_2_ be the two points in time at which we measure the degree of all the nodes, and we let *t*_*a*_(*i*) be an individual observation time for *i*=1,…,*N*, with *N* being the sample size. In general we may have *t*_*a*_(*i*)≠*t*_*a*_(*j*) for *i*≠*j*. We shall refer to the time period *t*_2_−*t*_*a*_(*i*) as Age, and as before the variable *X*_1_=*X*_1_(*v*)=*D*_2_−*D*_1_ is the increase in *v*’s degree from time *t*_1_ to *t*_2_.

**Fig. 3:**
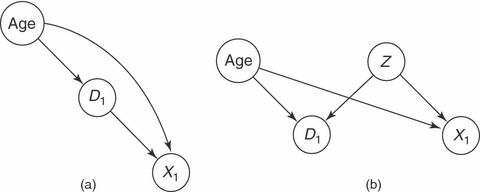
In both (a) a preferential attachment process and (b) a frailty process Age has a direct effect on *D*_1_: by the preferential attachment hypothesis, *D*_1_ has a direct effect on *X*_1_, whereas under the frailty hypothesis there is a confounder *Z* affecting both *D*_1_ and *X*_1_; furthermore, we assume that there is a direct effect of Age on *X*_1_

Fig. 3 shows the causal digraph in this case. Age clearly has an effect on *D*_1_. According to the preferential attachment hypothesis there is a direct effect of *D*_1_ on *X*_1_. In the frailty model there is no such direct effect, but there is instead a frailty variable *Z* which affects both *D*_1_ and *X*_1_. However, in Fig. 3 we have also included an arrow indicating a direct effect from Age on *X*_1_. Recall that *X*_1_ is the number of new connections that are acquired in a certain time interval. Although a direct effect of Age on *X*_1_ is captured neither in the Yule model nor in the frailty graph model, it is reasonable to expect such an effect in real world networks, such as sexual networks. In the previous test, it is easier to justify ignoring the effect of age altogether, since we may assume that *X*_1_ and *X*_2_ are two intervals which are relatively short and relatively close together, compared with the total time span of the graph process. In this case, however, we are in fact considering the entire time span from a node's birth until the present time, and the age effect is likely to be more pronounced. We should therefore take this effect into account.

**Fig. 4:**
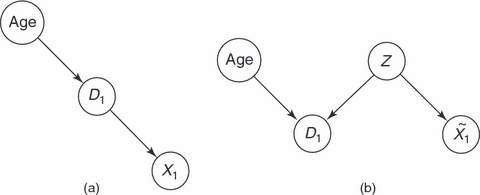
For both (a) a preferential attachment process and (b) a frailty process, we remove the direct effect of Age on *X*_1_ by transforming *X*_1_ into 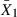

We shall assume that we know, or can estimate, the expected value and standard deviation of *X*_1_ given the age of the node. Thus, we have functions *f* and *s* such that *E*[*X*_1_(*v*)|Age(*v*)=*a*]=*f*(*a*) and var{*X*_1_(*v*)|Age(*v*)=*a*}=*s*(*a*)^2^. We define 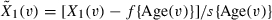 to be the standardized version of *X*_1_(*v*). If we now exchange *X*_1_ with 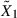 in Fig. 3, yielding Fig. 4, we can remove the edge from Age to 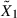. We can then use linear regression to estimate the effect of Age and *D*_1_ on 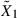, possibly by using a transformation *γ* on the dependent variable:


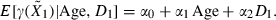
(13)

If the preferential attachment hypothesis is correct, *D*_1_ should have an effect on 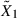, whereas Age should not, and we can expect to see *α*_2_ significantly different from 0, whereas *α*_1_ is not.

Let us assume instead that the frailty hypothesis holds. In this case, we induce a correlation between Age and *X*_1_ by controlling for *D*_1_. This can be seen from the causal diagram in Fig. 4, or by words in the following way: if *v* and *w* have the same degree at time *t*_1_, we can assume that the youngest node has the most favourable frailty variable, since it has spent the shortest time acquiring that degree. That node is therefore likely also to do better in the time interval from *t*_1_ to *t*_2_. If the frailty hypothesis holds, we therefore expect to see *α*_1_ significantly different from 0.

## 4. Numerical simulations

**Fig. 5:**
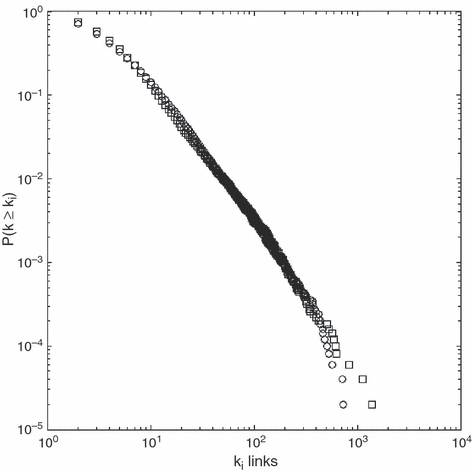
Cumulative degree distribution of the Yule graph (○) and the frailty graph (□): the size of the network is *N*=50000 nodes, and the parameters are *k*=1,*a*=0,*m*=2 and *Θ*=0.9985

We simulate in parallel graphs that are derived from the Yule process (Yule graphs) and frailty graphs from initial graph sizes *G*_0_ of 20 nodes, and with equal values of the parameters *a*,*m* and *k*_0_ as described in Section 2.7. The tail behaviour of the frailty graph is adjusted from equation (11), on the basis of the final graph size *N* and the scaling constant *q* of the corresponding Yule graph. The resulting graphs have similar scaling characteristics; Fig. 5.

**Fig. 6:**
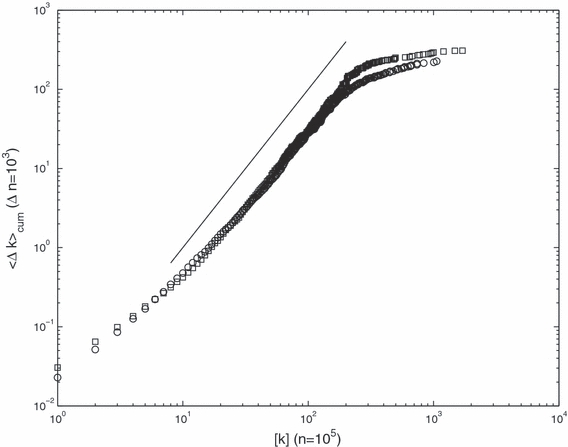
Cumulative mean number of links grouped by previous connectivity for a Yule graph (○) and a frailty graph (□): the rate of linkage is tested on networks of size *N*=10000 in an interval of Δ*N*=1000 nodes, and the model parameters are *k*=1,*a*=0,*m*=2 and *Θ*=0.999 (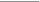, linear preferential attachment)

First we use the recommended graphical method by Jeong *et al.* (2003) to test for preferential attachment. In Fig. 6 the cumulative mean number of new links during a small time interval Δ*t* is plotted as a function of the connectivity at the beginning of the interval on log–log-axes. The mean numbers are group averages among all nodes with identical link numbers at Δ*t*=0. A line has been added showing the expected linear preference slope. It is clear that the two graphs are quite similar, implying that the method suggested cannot distinguish a random process acting on nodes with heterogeneous rates from true preferential attachment.

**Fig. 7:**
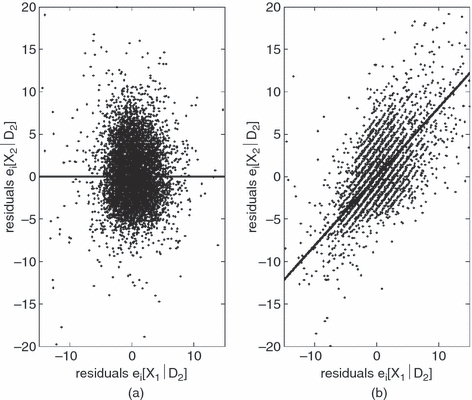
Partial regression plots of the residuals *e*_*i*_[*X*_2_|*D*_2_] on *e*_*i*_[*X*_1_|*D*_2_] for (a) the Yule graph and (b) the frailty graph: the slopes are equal to the *β*_1_-coefficients for *X*_1_ in the regression model equation (12); in the Yule graph *β*_1_=7.1×10^−3^, [−1.5×10^−2^;1.7×10^−2^], with the 95% confidence interval added in brackets; the corresponding value for the frailty graph is *β*_1_=8.1×10^−1^, [8.0×10^−1^;8.2×10^−1^]; simulations were performed with scaling factor *q*=2.5; the observation times are *N*_1_=27000, *N*_2_=40000 and *N*_3_=60000 nodes

Then, we test for preferential attachment as described in Section 3. Given three observations of the graphs, a linear regression of the number of new links in the second time period *X*_2_ is performed with the number of new links during the first time period *X*_1_ and the number of links at the start of the second time period *D*_2_ as explanatory variables. Fig. 7 shows partial regression plots and identifies the isolated effect of adding *X*_1_, where the slopes are identical to the regression coefficient *β*_1_ in the multiple-regression model (12). The test correctly identifies the preferential attachment process with *β*_1_ equal to 0. Thus, knowledge of *X*_1_ gives no further information about *X*_2_ once we have knowledge of *D*_2_. In the frailty graph, the *β*_1_-coefficient is significantly different from 0.

**Fig. 8:**
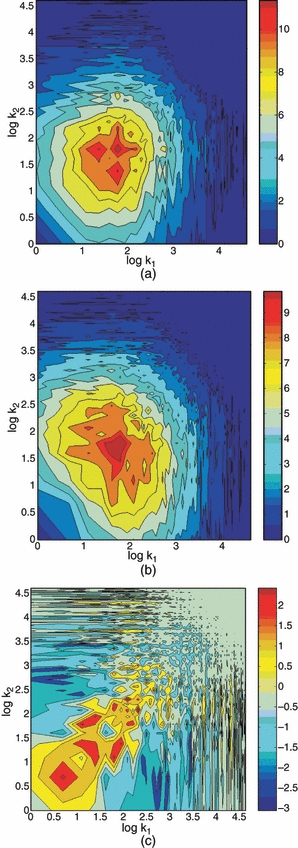
Joint degree distribution of (a) the Yule graph and (b) the frailty graph (the most frequent links in both graphs are links connecting medium degree nodes, producing an area with high frequency densities in the lower left-hand corners; high degree nodes are mostly connected to low degree nodes (bottom right and top left) and (c) the difference between the frailty graph joint degree distribution (the frailty-based topology has the largest density of links connecting medium degree nodes; the area with high density is extended in the Yule-based graph and stretched towards the axes)

The disparate linking dynamics in the preferential attachment graph and the frailty graph give rise to differences in other graph statistics. For example, the dynamics will affect the degree distribution in the neighbourhood of nodes of a given degree. The interconnection between nodes is commonly measured by the joint degree distribution *P*(*k*_1_,*k*_2_), describing the probability that a randomly selected link has end points in nodes with connectivity *k*_1_ and *k*_2_. It has the definition


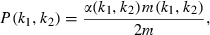
(14)

where *m* is the number of links connecting nodes of types *k*_1_ and *k*_2_, and *α*(*k*_1_,*k*_2_)=2 for *k*_1_=*k*_2_; otherwise *α*(*k*_1_,*k*_2_) is equal to 1 (Fig. 8).

## 5. Analysis on real network data

To demonstrate how the tests work, we used them on two sets of data. The first data set counts the number of citations of a set of comparable scientific publications obtained from the Thomson Reuters *Web of Knowledge* (). The second data set counts the number of sexual partners in a group of 18–49-year-old men. In the tables we report least squares estimates together with bootstrap estimates and confidence intervals from 10000 samples. In addition we also list the standardized estimates and bootstrap estimates with confidence intervals to allow for comparison of individual influence of the dependent variables.

## 5.1. Citation data

As our sample we used the 262 papers that were published in the mathematical journal *Random Structures and Algorithms* in the period 1998–2003, and we counted the citations up to and including 2008. The data were collected from the *Web of Knowledge*. The number of citations ranges from 0 to 79, with the mean being 7.16.

We let *t*_1_=2004, *t*_2_=2006 and *t*_3_=2008, and we let *D*_*i*_, for *i*=1,2,3, be the total number of times that a paper has been cited by the end of the year *t*_*i*_. Thus, we consider time intervals of 2 years: the value of *X*_1_ is the number of times that a paper has been cited in 2005 and 2006, whereas *X*_2_ is the number of times that it has been cited in 2007 and 2008.

**Fig. 9:**
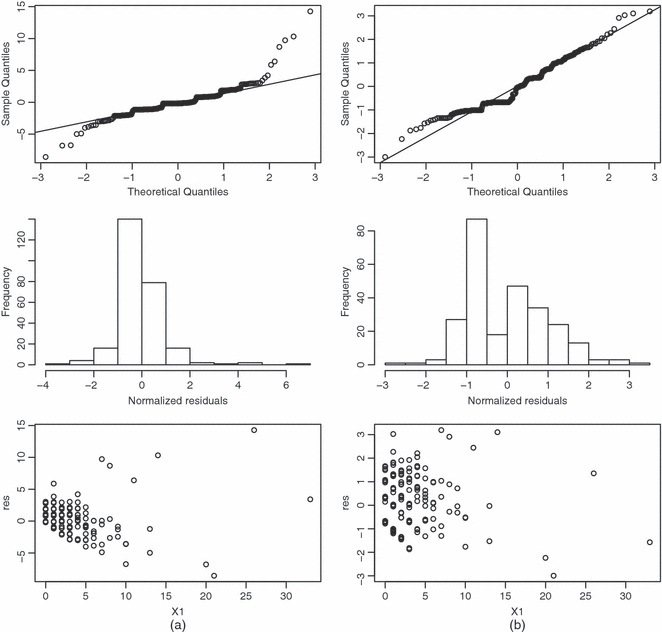
Analysis of the residuals: (a) untransformed; (b) Box–Cox transformation, *λ*=0.5

We first analyse the data by using the model that is defined by equation (12); the results are found in Table 1. In Fig. 9 we show the behaviour of the residuals of the linear regression when we use the untransformed data as the dependent variable, and when we use a Box–Cox transform with parameter 0.5. Using the untransformed data leads to residuals being clearly non-normal. Figs 9(d)–9(f) show that using transformed data leads to a better, albeit not perfect, fit to the normal distribution for the residuals, and we choose to use these variables in the linear regression.

**Table 1:** Analysis of model (12) for the citation data†

*Data*	*N*	*Variable*	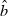	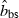	*95% confidence interval*	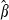	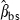	*95% confidence interval*	*Sign*
All	262	*X*_1_	0.29	0.29	(0.18,0.41)	0.71	0.71	(0.44,0.99)	0.000
All	262	*D*_2_	0.02	0.02	(−0.04,0.08)	0.10	−0.07	(−0.19,0.38)	0.255

† The table shows the unstandardized estimates 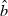 and bootstrap estimates 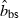 and standardized estimates 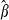 and bootstrap estimates 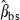, along with bootstrap confidence intervals.

Doing the regression analysis, we find that *X*_1_ has a strong and highly significant effect on *γ*(*X*_2_), whereas *D*_2_ does not. This gives us a strong indication that there is a frailty effect.

We now turn to the model that is defined by equation (13); the results here are found in Table 2. In this case it turns out that the logarithmic transformation 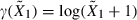 gives us the best approximation of the residuals to a normal distribution. In this case we find that there is an effect of Age on 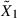, which, as explained in Section 3, also suggests that there is a frailty effect. In this case, however, the effect of *D*_1_ on 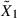 does not disappear, as in the previous test. This may be an indication that there is a certain preferential attachment effect as well.

**Table 2:** Analysis of model (13) for the citation data†

*Data*	*N*	*Variable*	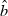	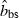	*95% confidence interval*	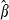	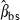	*95% confidence interval*	*Sign*
All	262	Age	−0.07	−0.07	(−0.11,−0.03)	−0.18	−0.18	(−0.28,−0.08)	0.000
All	262	*D*_1_	0.05	0.05	(0.045,0.063)	0.71	−0.72	(−0.62,0.86)	0.000

† The table shows the unstandardized estimates 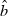 and bootstrap estimates 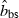 and standardized estimates 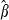 and bootstrap estimates 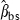, along with bootstrap confidence intervals.

## 5.2. Sexual partner network

In our second example we analyse data on numbers of lifetime sex partners in a group of 18–49-year-old heterosexual men, in addition to information on their partner numbers during the periods of 5, 3 and 1 year previously. The data are from a Norwegian survey that was conducted in 2002, and the partner distribution has previously been shown to be highly skewed (de Blasio *et al.*, 2007). We let *D*_1_ and *D*_2_ be the total number of partners 3 years and 1 year respectively before the study was conducted; let *X*_1_ and *X*_2_ be the numbers of new sexual partners in the first 2 years and the last 1 year of the 3-year period leading to the survey respectively. The variable *Y*_act_ (corresponding to Age in equation (13)) refers to the time since sexual debut counted in years.

The acquisition of sexual partners is a complex social phenomenon, depending on a person's age and marital (cohabiting) status as well as other factors. Thus, the age of sexual debut and the timing and duration of steady partnerships are likely to influence to a great extent the number of sex partners an individual will experience. To take these issues into account we also tested the short-term sexual pattern, where presumably changes in social–demographic factors are minor. In these analyses we let *D*_1−5_ and *D*_2−5_ be the total numbers of partners in the first 2 and 4 years with respect to the 5-year period leading to the survey.

The age of sexual debut among the men ranged from 9 to 30 years, and this makes the use of *Y*_act_ in equation (13) potentially problematic. The problem arises because we make an age adjustment for the dependent variable 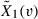 where we pool men with the same number of years of active sexual life, but with different biological age. Since sexual activity is known to decline with age, we should avoid having a large discrepancy between age and *Y*_act_. For this reason we include only those men who had their sexual debut between 15 and 19 years of age, who constitute 72% of the observations.

As with the citation data, we perform a Box–Cox transformation of the dependent variables *γ*(*X*_2_) and 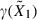. The results of the regression analyses that are defined by equation (12) argue for presence of frailty in the sexual partner network dynamics, in both longer and shorter periods of time. This is seen by the significant estimates of the *X*_1_-coefficients to predict *γ*(*X*_2_) when controlling for the absolute value of partners *D*_2_ and *D*_2−5_ (Table 3). Hence, the data suggest that individual variation may have an important influence on dynamics of sexual networks. There are also indications of preferential attachment, as seen from the significant effects of *D*_2_ and *D*_2−5_, particularly when conditioning on the total number of partners in the previous 5-year period. These results are in qualitative agreement with findings in a previous study where the same data (but different subsets) were fitted to a combined preferential attachment model with frailty (de Blasio *et al.*, 2007). In that study only the short-term dynamics gave significance to preferential attachment, and frailty was found to be necessary to produce an adequate model fit.

**Table 3:** Analysis of model (12) for sexual partnerships

*Data*	*Results for X*_1_					*Results for D*_2_				
		
	*N*	*λ*	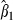	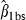	*95% confidence interval*	*Sign*	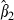	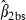	*95% confidence interval*	*Sign*
All	608	−0.62	0.32	0.33	(0.21,0.50)	0.000	0.12	0.13	(0.04,0.23)	0.003
20–29 years	144	−0.40	0.26	0.24	(−0.01,0.51)	0.012	0.03	0.08	(−0.14,0.45)	0.763
30–39 years	229	−0.81	0.19	0.24	(0.02,0.52)	0.007	0.30	0.28	(0.11,0.44)	0.000
40–49 years	220	−0.40	0.46	0.58	(0.36,1.08)	0.000	0.09	0.09	(−0.00,0.20)	0.139

	***Results for X*_1_**					***Results for D*_2−5_**				
		
	***N***	***λ***	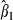	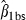	***95% confidence interval***	***Sign***	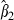	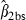	***95% confidence interval***	***Sign***
All	608	−0.63	0.20	0.21	(−0.03,0.45)	0.009	0.21	0.21	(0.01,0.47)	0.006
20–29 years	144	−0.43	0.06	0.06	(−0.28,0.42)	0.751	0.21	0.23	(−0.18,0.64)	0.238
30–39 years	233	−0.87	0.18	0.17	(−0.23,0.57)	0.078	0.23	0.26	(0.03,0.66)	0.026
40–49 years	231	−0.38	0.34	0.44	(0.07,1.05)	0.015	0.22	0.19	(−0.08,0.50)	0.008
Cohabiting	425	−1.63	0.20	0.21	(−0.03,0.45)	0.009	0.21	0.21	(0.01,0.47)	0.006
Single living	166	−0.07	0.31	0.30	(−0.06,0.61)	0.047	0.16	0.17	(−0.15,0.52)	0.312

The (5-year) analysis showed signs of preferential attachment among cohabiting men, but not among men who were living single at the time point when the survey was conducted. However, the latter group is heterogeneous in the sense that some of these men would have been engaged with a steady or cohabiting partner during the previous 5 years, whereas others would not. This factor could not be controlled for in the analysis. Instead, most of the cohabiting men (69%) had been living with the same partner for more than 5 years. In the second regression model that is defined by equation (13) we recover the same findings of presence of frailty and preferential attachment based on the significant coefficients of *Y*_act_ and *D*_1_ to predict 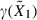 (Table 4).

**Table 4:** Analysis of model (13) for sexual partnerships

*Data*	*Results for Y*_act_					*Results for D*_1_				
		
	*N*	*λ*	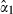	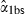	*95% confidence interval*	*Sign*	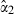	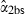	*95% confidence interval*	*Sign*
All	608	−0.65	−0.29	−0.29	(−0.37,−0.22)	0.000	0.21	0.21	(0.12,0.32)	0.000

In the light of the small sample size we should be cautious when interpreting the present findings. Preferably, the analyses should be repeated on larger data sets to test the consistency of the results. We also note that the present models are not adequate to make quantitative predictions, which will require more elaborate statistical methods.

## 6. Conclusion

In this paper, we have compared frailty random graphs with preferential attachment graphs. We have derived the frailty rate distribution for generating randomized power law networks, which reproduce the degree distribution of a Yule process. Generative network models commonly involve preferential attachment mechanisms, but often no effort is made to confirm proportionate growth from data. We have shown that randomized, heterogeneous growth processes exhibit spurious preferential attachment: nodes with large degree tend to acquire new links at a higher rate than other nodes, both in the frailty process and in the Yule process. Hence, the conjecture of preferential attachment cannot be tested from simple graphical plots.

We have set up two statistical tests for identifying frailty effects and preferential attachment mechanisms in evolving networks. The first is quite simple to perform, whereas the second requires an estimate of how the average rate of acquiring new edges evolves with the age of the nodes. The tests also require that we can find a transform of the dependent variable, which allows us to assume that the assumptions of the linear regression model are satisfied. Thus, they can generally be used to gauge potential frailty effects and preferential attachment before more sophisticated and time-intensive statistical methods are used. We have applied the tests to simulated data and real network data. From the latter analyses we conclude that the frailty may be a significant driving factor for the evolution of highly skewed networks. However, there are cases where the preferential attachment mechanism seems well grounded. The prime example is the well-studied World Wide Web network as discussed in Dorogovtsev and Mendes (2003), where advanced search engines make direct use of previous rates of linking to rank retrieved information. For most other types of networks, particularly those with a more limited natural scale, inherent heterogeneity between nodes is potentially of great importance and should not be neglected.

The frailty model that is suggested here is highly simplistic and does not reproduce important structures like local clustering, which is observed in natural networks. A model that is intended to be a realistic approximation of real world networks should also take such phenomena into account. However, the purpose here has not been to make realistic network models, but to investigate whether the frailty hypothesis can be a reasonable contender for a growth mechanism, when compared with preferential attachment, which has until now been a very popular model in much of the literature.
